# Pharmacological Reactivation of the Silenced *FMR1* Gene as a Targeted Therapeutic Approach for Fragile X Syndrome

**DOI:** 10.3390/brainsci9020039

**Published:** 2019-02-12

**Authors:** Daman Kumari, Inbal Gazy, Karen Usdin

**Affiliations:** Section on Gene Structure and Disease, Laboratory of Cell and Molecular Biology, National Institute of Diabetes, Digestive and Kidney Diseases, National Institutes of Health, Bethesda, MD 20892, USA; inbal.gazy@nih.gov (I.G.); ku@helix.nih.gov (K.U.)

**Keywords:** fragile X syndrome, gene reactivation, RNA:DNA hybrid, FMRP, histone methylation, DNA methylation, *FMR1*, PRC2

## Abstract

More than ~200 CGG repeats in the 5′ untranslated region of the *FMR1* gene results in transcriptional silencing and the absence of the *FMR1* encoded protein, FMRP. FMRP is an RNA-binding protein that regulates the transport and translation of a variety of brain mRNAs in an activity-dependent manner. The loss of FMRP causes dysregulation of many neuronal pathways and results in an intellectual disability disorder, fragile X syndrome (FXS). Currently, there is no effective treatment for FXS. In this review, we discuss reactivation of the *FMR1* gene as a potential approach for FXS treatment with an emphasis on the use of small molecules to inhibit the pathways important for gene silencing.

## 1. Introduction

Fragile X syndrome (FXS, MIM 300624) is the most common form of inherited cognitive disability affecting 1 in 5000 males and 1 in 8000 females [1,2]. In addition to intellectual disability, FXS often presents with a characteristic behavioral and physical phenotype that includes attention deficit, anxiety, and autism spectrum disorders as well as a prominent forehead, long face, and protruding ears [3]. FXS is caused by the loss of function of the fragile X mental retardation protein (FMRP) that is encoded by the fragile X mental retardation 1 (*FMR1*) gene. An unstable CGG repeat tract is present in the 5’ untranslated region (UTR) of the *FMR1* gene. In the general population, the repeat tract has less than 45 repeats [4]. *FMR1* alleles with 55–200 repeats are known as premutations (PM), and those with greater than 200 CGG repeats are referred to as full mutations (FM) [5,6]. Most FM alleles show aberrant DNA methylation and are transcriptionally silenced, resulting in the absence of FMRP and thus FXS [7,8]. A minority of FXS patients who do not carry the FM have deletions or point mutations in critical regions of FMRP that result in a loss of function [9,10,11,12]. Some FXS patients have a mixture of PM and FM alleles and/or some proportion of unmethylated FM alleles. These individuals make some FMRP and present with a milder clinical phenotype [13,14,15,16,17,18,19,20,21,22]. 

FMRP is an RNA-binding protein that regulates the transport and translation of many mRNAs in the brain [23,24,25,26,27]. The loss of FMRP results in defects in synaptic plasticity and neuronal development [28,29]. In addition, studies have implicated FMRP in the cellular stress response [30], cancer metastasis [31], the DNA damage response [32,33], pre-mRNA alternative splicing [34], and RNA editing [35,36]. Thus, the loss of FMRP has pleiotropic effects. 

**Figure 1:**
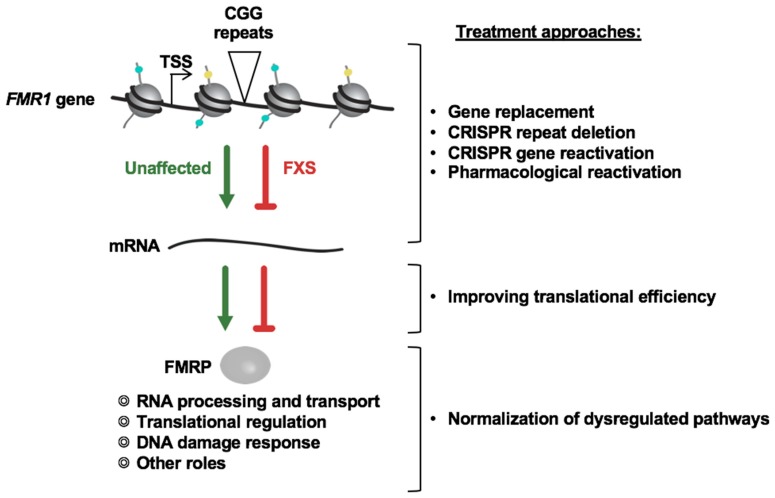
Possible treatment approaches for fragile X syndrome (FXS).

There is no cure or effective treatment for FXS. Most available medications provide only symptomatic relief, are not very effective, and can be associated with deleterious side effects. Two different options for developing an effective treatment for FXS are possible: (i) compensating for the loss of FMRP function by identifying and normalizing the altered pathways, and (ii) restoring FMRP expression either by reactivating the silenced *FMR1* gene or by providing exogenous FMRP using gene therapy or mRNA-based approaches (Figure 1). While preclinical testing of targeted treatment strategies aimed at compensating for the loss of FMRP has been successful in mouse models of FXS (reviewed in [37]), many of the clinical trials based on these studies were unsuccessful (see [38] for a recent review). There are a variety of possible explanations for why this was the case, including heterogeneity in the FXS patient population, the lack of suitable objective outcome measures, and the fact that only a subset of altered pathways were targeted. 

In principle, restoring FMRP expression may be more broadly useful as it targets the root cause of the disease, the absence of FMRP. Different strategies are being pursued for this purpose. Preliminary studies using clustered regularly interspaced short palindromic repeats (CRISPR)/Cas9-mediated gene editing approaches to (i) delete the expanded CGG repeats in FXS patient cells [39,40], (ii) induce DNA demethylation in the *FMR1* promoter region [41], and (iii) target transcriptional activators to the *FMR1* promoter in FXS cells [42] have all been successful in partially reactivating the *FMR1* gene in cell models. Gene therapy approaches are also being pursued to restore FMRP expression. For example, FMRP expression can be achieved in the brains of *Fmr1* knockout (KO) animals using adeno-associated virus (AAV) vectors for gene delivery. Such exogenous expression of FMRP corrects abnormally enhanced hippocampal long-term synaptic depression [43] and reverses some of the abnormal behaviors seen in this mouse model [44]. These approaches are discussed elsewhere in this special issue. In this review we will focus on pharmacological approaches for *FMR1* gene reactivation [45,46,47,48]. The use of small molecules for gene reactivation is currently being tested for a number of other disorders including myelodysplatic syndromes [49], Rett Syndrome [50,51], Angelman syndrome [52], frontotemporal dementia [53], and Friedreich ataxia [54]. As a result, the list of small molecules able to reactivate silenced genes that have been approved for use in humans is growing rapidly [55]. The search for small molecules suitable for gene reactivation can be divided into two categories: (i) a rational or candidate approach, in which specific pathways important for silencing are identified and targeted for gene reactivation, and (ii) an unbiased screening approach to identify small molecules that are capable of reactivating the silenced gene in patient cells. 

## 2. Targeting Specific Pathways and Proteins Involved in *FMR1* Gene Silencing in FXS

The rational or candidate approach to reactivating the *FMR1* gene in FXS requires a clear understanding of the underlying silencing mechanism. Despite the fact that it has been more than 25 years since the *FMR1* gene and the causative CGG expansion mutation were identified, the mechanism by which the repeat expansion leads to gene silencing in FXS is still not completely understood. In the following sections, we will review the research that has identified some of the epigenetic modifications present on silenced alleles, some of the proteins important for these modifications, and the various small molecule-based approaches that have been used to date for gene reactivation.

### 2.1. Epigenetic Marks Associated with the Silenced FMR1 Gene in FXS

The transcriptional activity of a gene is regulated by various epigenetic marks that include DNA methylation and modifications of the N-terminal tails of histone proteins associated with the promoter. In general, transcriptionally active regions are hypomethylated and enriched for acetylated histones. These modifications result in an open chromatin conformation or euchromatin. In contrast, transcriptionally inactive regions often show high levels of CpG methylation of the DNA and are associated with histone modifications that result in compact chromatin (heterochromatin). Heterochromatin is generally hypoacetylated and enriched for H3 and H4 histones methylated at specific lysine residues. Some of these modifications are characteristic of facultative heterochromatin, which is found on developmentally silenced genes, whilst other modifications are more typical of constitutive heterochromatin, which is important for the silencing of repeat elements in the genome. 

Most of the knowledge about the contribution of various epigenetic modifications to *FMR1* gene silencing has been obtained from studies done with FXS patient cells. It was observed early on that the FM alleles show increased CpG methylation [7,8]. In vitro methylation of the *FMR1* promoter repressed its activity in transient expression assays [56], perhaps because it abolishes the binding of the transcription factor alpha-Pal/Nrf-1 and reduces binding of upstream stimulatory factor (USF) 1 and USF2 [57]. Moreover, treatment of FXS patient cells with an inhibitor of DNA methyltransferase 1 (DNMT1), 5-azadeoxycytidine (5-aza-dC), leads to gene reactivation [45]. These data, together with the existence of rare individuals with unmethylated FM alleles who are high-functioning, reinforce the importance of DNA hypermethylation and *FMR1* gene silencing for the development of FXS symptoms. 

The *FMR1* promoter in FXS patient cells is also associated with a decrease in the levels of active histone marks that include acetylation of histone H3 at lysine 9 (H3K9ac), di-methylation of lysine 4 (H3K4me2), and acetylation of histone H4 at lysine 16 (H4K16ac) [46,47,58]. Moreover, the levels of repressive histone marks are increased on the FM alleles. These include di- and tri-methylation of histone H3 at lysine 9 (H3K9me2, H3K9me3), tri-methylation of lysine 27 (H3K27me3), and tri-methylation of histone H4 at lysine 20 (H4K20me3) [58,59,60]. Thus, the silenced allele is enriched for histone modifications characteristic of both facultative and constitutive heterochromatin.

### 2.2. Models for FMR1 Gene Silencing

In addition to the identification of chromatin modifications important for silencing, a knowledge of the timing and sequence of events leading to these modifications may also be important for designing effective strategies for gene reactivation. Early studies of FXS embryonic stem cells (ESCs) suggested that H3K9me2 is a relatively early event in the silencing process, occurring before DNA methylation [61]. This is consistent with the observation that DNA demethylation does not affect the levels of H3K9 methylation in differentiated cells [58,62]. Many regulators of heterochromatin formation bind methylated histone lysines [63] and recruit DNA methyltransferases. For example, methylated H3K9 serves as a binding platform for the recruitment of heterochromatin protein 1 (HP1) that in turn recruits the de novo DNA methyltransferases 3A and 3B [64]. Enhancer of Zeste 2 (EZH2), the catalytic component of polycomb repressive complex 2 (PRC2) that is responsible for H3K27me3, has also been shown to be necessary for DNA methylation of PRC2-target genes [65], although it is not sufficient for methylation at all loci [66]. Since DNA demethylation of FXS alleles results in increased H4K16 acetylation [47], it may be that H4K16 deacetylation occurs downstream of DNA methylation. However, a better understanding of the silencing process is required in order to understand the relationships between all of the epigenetic factors involved.

One model for gene silencing suggests that silencing is initiated by the loss of binding of an insulator protein to a region upstream of the *FMR1* promoter [67]. This in turn is suggested to disrupt the chromatin boundary upstream of the *FMR1* promoter, thus allowing the spread of DNA methylation and repressive histone marks from an upstream heterochromatic zone [60,67]. A recent study has also suggested that CCCTC-binding factor (CTCF) plays a role in maintaining the topologically associated domains (TADs) at disease-associated short tandem repeats like those at the *FMR1* locus and that disruption of the higher-order genome folding alters the enhancer landscape leading to gene silencing [68]. While CTCF binding has been seen in some cells from unaffected individuals and is missing from the same cell type in affected individuals [69,70], demethylation of the *FMR1* promoter in FXS lymphoblastoid cells by 5-aza-dC treatment did not restore CTCF binding. Furthermore, knockdown of CTCF in cells from unaffected individuals and those with unmethylated FMs did not result in the spreading of DNA methylation from the upstream boundary into the *FMR1* promoter [70]. This suggests that CTCF does not act as an insulator at the *FMR1* locus. Whether the loss of the chromatin boundary is relevant to *FMR1* gene silencing is still unclear. 

An alternate hypothesis is that silencing is initiated by chromatin changes occurring in the repeat itself. This idea is supported by the observation that the constitutive heterochromatin marks, H3K9me3 and H4K20me3, show a focal distribution on the *FMR1* gene in FXS cells, being enriched in the vicinity of expanded CGG repeats. In contrast, the facultative heterochromatin marks, H3K9me2 and H3K27me3, are more widely distributed, perhaps, due to the propensity of these marks to spread [60]. In this view, the spreading of the facultative heterochromatin from the vicinity of the expanded repeat results in its merging with the upstream heterochromatin zone, and thus giving the impression of a loss of boundary function. 

**Figure 2:**
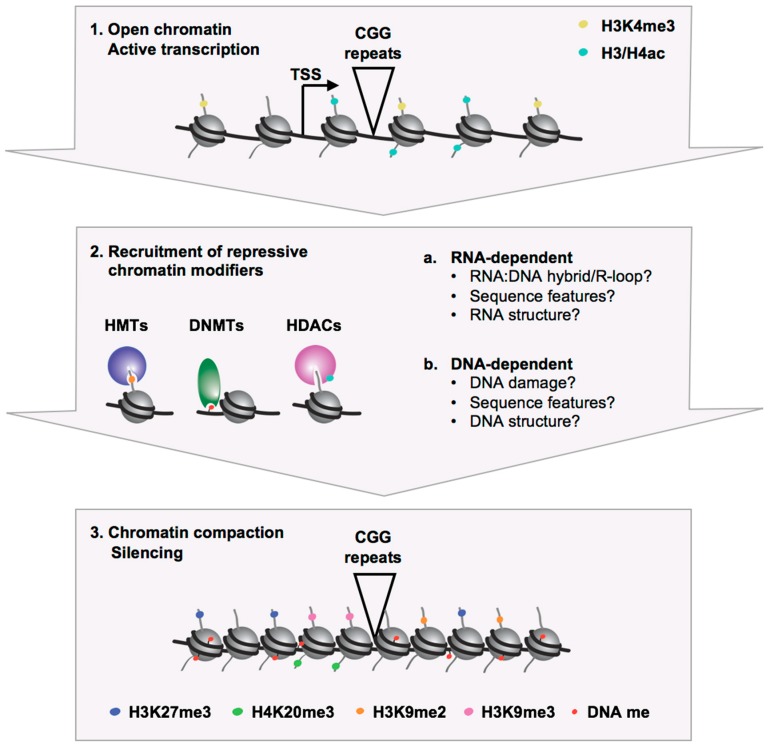
Schematic representation of the recruitment of repressive chromatin modifiers to the *FMR1* locus leading to a heterochromatic silenced state in FXS.

The expanded CGG repeats could trigger heterochromatin formation by processes that are DNA-or RNA-dependent (Figure 2) (reviewed in [71]). The expanded CGG/CCG repeats in the DNA and RNA form unusual structures in vitro that include stem-loop/hairpins, G-tetraplexes/quadruplexes, and R-loops/RNA:DNA hybrids [48,72,73,74,75,76,77,78,79,80,81,82,83,84,85,86], and there is evidence that such structures are also formed in vivo [84,86]. These secondary structures may in turn recruit chromatin modifiers. For example, it has been suggested that CGG hairpins can directly recruit DNA methyl transferases [73,87]. Sequence-specific factors that bind CGG repeats in the DNA may also play a role in heterochromatization of the *FMR1* locus by directly recruiting chromatin modifiers, as has been reported for Suv39h recruitment to major satellite repeats in mice [88]. 

An RNA-based mechanism for the initiation of silencing is appealing since it has been shown that the increase in the levels of repressive mark H3K27me3 after 5-aza-dC treatment is dependent on the levels of the *FMR1* transcript and that blocking deposition of this repressive mark is able to significantly delay the re-silencing that happens after 5-aza-dC is withdrawn [48,62]. In addition, the observed similarity between the chromatin marks associated with the silenced *FMR1* gene and *Sat2* repeats [60] suggests that *FMR1* gene silencing might involve a mechanism similar to the RNA-dependent mechanism involved in the formation of pericentromeric heterochromatin (reviewed in [89]). Both RNAi-dependent and RNAi-independent RNA-based models have been proposed for silencing in FXS. RNAi-dependent gene silencing involves the generation of small double stranded (ds) RNAs by Dicer cleavage of larger dsRNAs. The small dsRNAs associate with Argonaute (AGO) proteins which in turn recruit chromatin modifiers to the locus that result in transcriptional silencing. A complex mixture of sense and antisense transcripts have been identified at the *FMR1* locus, and their pairing could provide a substrate for the generation of small dsRNAs [60,69,90]. Another potential source of small dsRNAs could be the hairpins formed by the CGG repeats in the RNA which are known to be substrates for Dicer [80]. Indeed, small RNAs, ~20 nucleotide (nt) in length that are derived from the *FMR1* promoter and CGG repeat region, have been reported in FXS lymphoblastoid cells after treatment with 5-aza-dC. However, similar levels of these small RNAs were also seen in cells from unaffected individuals [60]. A recent study also reported the presence of ~21 nt RNAs containing CGG repeats in association with AGO1 in FXS ESCs. This was suggested to lead to the recruitment of the H3K9 histone methyl transferase, SUV39H, to the *FMR1* locus [91]. However, while members of the AGO protein family have been shown to be important for heterochromatin formation and transcriptional silencing of repetitive sequences in fission yeast [92], *Tetrahymena* [93,94] and *Drosophila* [95], their role in transcriptional gene silencing in mammals in general [96,97,98] and in the context of FXS in particular [82] is unclear. 

RNAi-independent mechanisms for heterochromatin formation at the *FMR1* locus could involve the recruitment of chromatin modifiers by the *FMR1* mRNA through a mechanism similar to that used by long non-coding RNAs like *HOTAIR* [99], *Air* [100], and *Xist* [101,102]. This would require the interaction of *FMR1* mRNA with the *FMR1* locus either directly or indirectly. The *FMR1* mRNA from reactivated alleles is preferentially enriched in the chromatin fraction and thus supporting such interaction [48]. Moreover, an R-loop is present at the *FMR1* locus [48,82,83,84,86]. R-loops can potentially recruit chromatin modifiers to a genomic locus, as was proposed for the recruitment of the PRC2 responsible for H3K27me3 deposition at the *RASSF1A* gene [103]. Stable R-loops have also been shown to recruit the G9a histone methyltransferase, which is responsible for H3K9me2, to expanded GAA repeats in the *frataxin* gene in Friedreich ataxia [83]. Furthermore, RNA:DNA hybrid formation has also been implicated in the recruitment of H3K9 trimethylases, Suv39h1 and Suv39h2, to the heterochromatin in mouse ESCs [104]. While an R-loop is present at the *FMR1* locus in unaffected individuals, the R-loop formed on FM alleles would be longer and more stable and thus perhaps more effective at recruitment of epigenetic repressors. PRC2 can directly bind quadruplexes in RNA or unstructured G-rich RNA sequences [105] and has been shown to interact with the *Fmr1* transcript in mouse ESCs [106]. Since the 5′ end of mouse and human *FMR1* transcripts share much sequence similarity, it is possible that PRC2 also directly binds the human *FMR1* transcript. This may represent one way in which R-loops are able to recruit PRC2 to the 5′ end of the *FMR1* gene. In addition, SUV39H1 has also been shown to directly bind RNA in both mice and humans, although it does not show any major preference for particular structures or sequences [104,107,108,109]. A persistent R-loop could also cause transcription termination. The resulting short nascent RNAs from the 5’ end of the *FMR1* transcript may also have the potential to recruit chromatin modifiers including PRC2 [110]. 

### 2.3. Targeting the Activity of Repressive Chromatin Modifiers for Gene Reactivation 

In principle, inhibition of the repressive epigenetic modifiers involved in the silencing of FM alleles could result in gene reactivation. For example, inhibition of DNMT1 with 5-aza-dC reactivates the *FMR1* gene in FXS lymphoblastoid, fibroblasts, and induced pluripotent stem cell (iPSC)-derived neural progenitor cells (NPCs). Given that differentiated cells express little, if any, active demethylation factors, DNMT1 inhibitors are thought to be effective primarily in dividing cells where methylation of nascent daughter strands is prevented and existing methylation marks are gradually diluted by successive rounds of replication. In addition to 5-aza-dC, another somewhat less effective DNMT1 inhibitor, 5-azacytidine (5-aza-C), has been shown to partially reactivate the *FMR1* gene in NPCs derived from FXS iPSCs [111,112] and in vitro differentiated neurons [111]. However, given that only ~50% of the cells in the differentiated population were neuronal cells, whether demethylation actually occurred in the neurons is unclear. An effect of DNMT1 inhibitors in non-dividing cells like neurons would be both surprising and important given the need to restore FMRP expression for proper neuronal function. Valproic acid (VPA), a drug used for the treatment of epilepsy and as a mood stabilizer, has been shown to trigger replication-independent active demethylation at other loci [113]. However, treatment of FXS lymphoblastoid cells with VPA had little effect on transcriptional activation and did not induce DNA demethylation of the *FMR1* gene [114]. Another compound that has been suggested to cause DNA demethylation is methotrexate (MTX), an inhibitor of dihydrofolate reductase [115,116]. However, while treatment of FXS fibroblasts with MTX did indeed reactivate the *FMR1* gene, it did not decrease promoter methylation, suggesting that its effect on *FMR1* transcription was independent of local DNA demethylation [117]. There is some evidence to suggest that 5-aza-dC can affect gene expression independently of its effects on DNA demethylation, and it is possible that this accounts for the high efficacy of this compound relative to other DNMT1 inhibitors [118,119].

Treatment of FXS cells with inhibitors of class I, II, and IV histone deacetylases (HDAC) including 4-phenylbutyrate, sodium butyrate (NaB), trichostatin A (TSA), romidepsin, and vorinostat have been shown to be ineffective at reactivating the silenced *FMR1* gene; however, a synergistic effect of 5-aza-dC and some of these inhibitors has been reported [120,121]. Treatment with either NaB or TSA increased total H4 acetylation but did not increase H3 acetylation [58]. In contrast, inhibition of Sirtuin 1(SIRT1), a class III HDAC, is able to reactivate the *FMR1* gene to levels similar to those seen with 5-aza-dC treatment [47] and results in increased acetylation at both H3 lysine 9 and H4 lysine 16. Because SIRT1 inhibitors do not require replication to be effective, this class of inhibitors might be better for the reactivation of FM alleles in non-dividing cells like neurons. 

While gene reactivation with 5-aza-dC treatment does not lead to any significant changes in the levels of repressive histone marks H3K9me2, H3K9me3, or H4K20me3 [58,62], it increases the levels of H3K27me3 as well as EZH2, the catalytic component of the PRC2 that is responsible for H3K27me3, at the *FMR1* promoter [62]. EZH2 inhibition by itself does not reactivate the *FMR1* gene in FXS cells; however, EZH2 inhibitors are very effective at preventing the re-silencing of reactivated alleles after 5-aza-dC is withdrawn [48]. This would be consistent with the idea that PRC2 recruitment to the *FMR1* locus likely also occurs prior to DNA methylation. Since DNA methylation is likely to be clonally propagated in differentiated cells [122,123], DNA methylation effectively provides an epigenetic memory of the silenced state in these cells. Thus, inhibition of DNMT1 is likely to be required for inhibitors of early steps in the silencing pathway to be effective at reactivation of silenced alleles. 

It is also important to note that the efficacy of all of these compounds varies considerably between different cell lines. Whether this is due to differences in the length of the CGG repeat tract and the extent of DNA methylation or other genetic differences that might impact the drug uptake and efflux is unclear. Furthermore, these compounds are likely to have off-target effects that include altered expression of other repressed genes [124,125] and other effects on cell viability [126,127].

### 2.4. Targeting the Recruitment of Chromatin Modifiers for Gene Reactivation

While it has been reported that some inhibitors of epigenetic modifying enzymes do not have the global effects on gene expression that one might expect [124,125], in principle, a more gene-specific strategy for the reactivation of *FMR1* would reduce the likelihood of undesirable effects at other loci. Given that inhibition of PRC2 after 5-aza-dC withdrawal prevents the re-silencing of reactivated alleles and that the *FMR1* transcript is involved in PRC2 recruitment and gene silencing, blocking this recruitment might be one way to achieve a more specific effect on *FMR1* expression. This could be accomplished either by preventing the interaction of *FMR1* mRNA with the *FMR1* locus or by blocking the binding of *FMR1* mRNA to PRC2. For example, Compound 1a (9-hydroxy-5, 11-dimethyl-2-[2-(piperidin-1-yl)ethyl]-6H-pyrido[4,3-b]carbazol-2-ium) is a small molecule that stabilizes the hairpins formed by the CGG repeats in the RNA (rCGG) and disrupts protein-binding [128,129]. Treatment of FXS lymphoblastoid cells with Compound 1a reduced H3K27me3 levels and prevented re-silencing of 5-aza-dC-reactivated alleles [48]. However, this treatment had no effect on the formation of RNA:DNA hybrids, suggesting that Compound 1a was acting to prevent re-silencing by blocking the interaction of *FMR1* mRNA with PRC2 [48]. Since Compound 1a also binds other G-rich repeats [130], and such repeat tracts are present at multiple locations in the human genome, it is possible that Compound 1a will have undesirable effects on the expression of other genes. While the extent of off-target effects of Compound 1a requires additional study, the development of more *FMR1*-specific small molecules that are also able to block PRC2 recruitment may be desirable.

## 3. Unbiased High-Throughput Screens to Identify Compounds that Reverse *FMR1* Gene Silencing

High-throughput screening (HTS) is an approach to accelerate drug discovery that involves testing a large number of potential biological modulators and effectors against a specific target. With respect to identifying compounds that can reactivate the *FMR1* gene in FXS cells, HTS can help identify new biologically active small molecules against known targets, for example, DNA methyltransferases, histone deacetylases, and histone methyltransferases, as well as identify additional targets that might provide new insights into the gene silencing mechanism. This is particularly important as many of the compounds that have been shown to reactivate the silenced *FMR1* gene so far are toxic and may not be suitable for long-term use in humans.

The success of an HTS depends on a robust readout that can be measured using an assay that is reliable and economical. In addition to the detection assay, the cells used in the screen are also important. Most FXS fibroblasts are mosaic for PM and FM alleles as well as their methylation status. Hence, it is difficult to assess if the increase in FMRP levels is due to an increase in translation from transcriptionally active alleles or reactivation of the silenced alleles. Furthermore, primary fibroblasts have a finite life span making it difficult to generate the large number of cells required for HTS. These characteristics make primary human fibroblast cells less desirable for HTS. In contrast, FXS iPSC-derived neural stem cells (NSCs) or NPCs are suitable for HTS because they can be used to rapidly generate a large number of cells. Furthermore, given that FM alleles in FXS iPSCs remain methylated [131,132], it is relatively easy to generate lines with a homogeneous population of silenced FM alleles. 

In this respect, HTS designed for identifying drugs that can increase *FMR1* transcription have relied on both the detection of endogenous FMRP in FXS NSCs or NPCs and the use of reporter cell lines. In the following sections we review the library screens that have been done thus far to identify compounds that reactivate the *FMR1* gene.

### 3.1. HTS Based on Measuring Endogenous FMRP Levels

Two different HTS have been reported for identifying molecules that are able to reactivate the *FMR1* gene [133,134]. For one of these screens, a specific and sensitive time-resolved fluorescence resonance energy transfer (TR-FRET)-based FMRP assay was developed and used to perform HTS in a 1536-well plate format [134]. Given that long CGG repeats negatively affect translation, the authors chose an iPSC line carrying a completely methylated FM allele with relatively short CGG repeats (~300) to generate NSCs and neurons. The NSCs were treated with 5-aza-dC to confirm the production of FMRP by western blot analysis and TR-FRET assay. FXS NSCs and in vitro differentiated neurons were first evaluated in test screens of a LOPAC^1280^ compound library, and two hits were identified, protoporphyrin IX and SB216763. These hit compounds were further validated by the dose response in TR-FRET assay and in a quantitative reverse transcription (qRT)-PCR assay for *FMR1* mRNA. The authors then screened a ~4000 compound FDA-approved library. Four additional compounds (sodium decanehydroxamate, geliomycin, tibrofan, and deserpidine) were identified from this HTS. With the exception of sodium decanehydroxamate, which is known to have HDAC activity, the mode of action of these compounds is unknown. While the compounds identified in this HTS were effective at very high concentrations and thus not likely to be biologically useful, it provides proof of principle of the approach and suggests that better lead compounds might be identified using larger compound libraries. 

In another screen, Kaufmann et al. [133] used high-content imaging with FMRP antibodies to screen 50,000 compounds in a 384-well plate format. The cells used were FXS NPCs derived from an iPSC line carrying a single methylated *FMR1* allele with ~480 CGG repeats. The authors also used 5-aza-dC as a positive control compound for the HTS. A total of 2099 compounds were identified that induced a small FMRP increase, and 790 of those were further tested in dose response assays. Only one compound was identified that had a previously known mode of action—a hydroxamate-based HDAC inhibitor, similar to the one identified in the HTS by Kumari and Swaroop et al. [134]. The identity of other hit compounds was not disclosed. The advantage of high-content imaging-based FMRP detection is that it provides information about FMRP levels in single cells. However, careful calibration is required to enable the detection of weak hits. 

### 3.2. HTS Using Knock-In Reporter Cell Lines

While HTS based on assays using antibodies to detect FMRP were successful at identifying a number of hit compounds, the cost of antibodies is a limiting factor. To eliminate the requirement for antibodies, Li et al. [112] inserted the Nano luciferase gene (Nluc) into the endogenous *FMR1* gene locus in FXS iPSCs and control H1 ESCs using CRISPR/Cas9 gene editing. The authors then generated NPCs from the H1 control and FXS *FMR1*-Nluc reporter lines and optimized the HTS in 384-well and 1536-well plates. Both 5-aza-C and 5-aza-dC were used as positive control compounds to screen a 128 epigenetic compound library and ~1134 FDA-approved drug library using NPCs differentiated from the FXS-*FMR1*-Nluc reporter iPSCs. While no new compounds were identified in these screens, the sensitivity and cost-effectiveness of these reporter lines will make it feasible to screen very large compound libraries.

The FMRP-based screening assay and the Nano-luc reporter lines share a common drawback—namely, the negative effect of CGG repeats on translation, which limits the amount of protein that can be detected. Thus, these assays are less sensitive than assays that detect *FMR1* mRNA levels. However, currently, RNA detection assays are not economical, and the generation of new reporter cell lines that allow the effect of compounds on transcription or translation to be distinguished would thus be highly desirable for large library screens.

## 4. Limitations and Challenges of Using *FMR1* Gene Reactivation as a Treatment Approach for FXS

Restoring FMRP expression in FXS cells by reactivating the endogenous *FMR1* gene is a potentially useful treatment option for FXS. However, many challenges to this approach remain. More work is needed to identify additional proteins important for gene silencing that can be targeted for gene reactivation. Conducting genome-wide CRISPR/siRNA screens for gene reactivation is one way to identify new targets. In addition, better model systems to understand the initiating events leading to gene silencing may allow the identification of novel proteins for pharmacological targeting. Early work in humans had led to the suggestion that *FMR1* gene silencing occurs relatively late in embryonic development at around 10 weeks of gestation [135]. If so, then the FM alleles in ESCs would be expected to be active, thus making these cells useful for understanding early steps in the silencing process. Indeed, a few studies have reported that the *FMR1* gene is actively transcribed in FXS ESCs and undergoes differentiation-induced silencing [61,82,136]. However, many FXS ESC lines already show some DNA methylation, suggesting that differentiation *per se* is not required for gene silencing [137,138]. Moreover, the silenced *FMR1* gene was not reactivated in iPSCs derived from FXS patients [131,132]. Both human ESCs and iPSCs are thought to more closely resemble primed pluripotent stem cells rather than the earlier more naïve state present in the preimplantation embryo, and it may be that *FMR1* gene silencing occurs at an earlier stage of embryonic development. It is therefore possible that naïve FXS iPSCs or ESCs could provide a better cell model for understanding the very earliest events in *FMR1* gene silencing [139]. 

Additional challenges include the necessity for overcoming the negative effect of the CGG repeats on the translation efficiency of *FMR1* mRNA [140,141,142,143] and to reduce the toxicity associated with rCGG expression that is thought to be responsible for fragile X-associated tremor/ataxia syndrome (FXTAS) and fragile X-associated primary ovarian insufficiency (FXPOI) in PM carriers [144,145,146,147,148]. Indeed, some carriers of unmethylated FM alleles have been reported to show symptoms of FXTAS [149,150,151]. However, there is a wide variability in the expression levels of FMRP in unaffected individuals [152] and FM females who express FMRP in only 50% of their cells usually present with milder intellectual impairment [153,154]. Similarly, males mosaic for PM and FM alleles and those with unmethylated FM alleles can make some FMRP and can be high-functioning. Thus, reactivation that results in expression of even low levels of FMRP may be clinically beneficial. In principle, pharmacological approaches that maximize translation may be useful in reducing the level of *FMR1* gene reactivation required. This would also reduce the risk of developing pathology resulting from rCGG expression. Furthermore, small molecules like Compound 1a that prevent gene re-silencing in a relatively gene-specific way while also reducing the deleterious effects of rCGG may be particularly useful [48,128,129]. 

Finally, the challenge of if, when, and how a gene reactivation approach could be deployed needs to be considered. For families with a known history of FXS, preimplantation genetic diagnosis might be the preferred option [155]. Most new cases of FXS are diagnosed when the child is already 2–3 years old [156]. While FXS deficits that likely arise during embryonic life are unlikely to be modulated by postnatal gene reactivation, there is evidence from work in *Fmr1* KO mice to suggest that increasing FMRP production during postnatal life may still have some clinical benefit [157,158]. Whether gene reactivation in utero will be feasible given the potentially detrimental effects of epigenetic modulators in early development remains to be seen.

## 5. Concluding Remarks

Preliminary studies using cell-based models for FXS have shown that it is possible to reactivate the silenced *FMR1* gene and suggested approaches for gene reactivation that are most likely to be effective. However, we still have a long way to go before this approach is therapeutically useful. A number of new tools are needed, including an animal model that recapitulates the repeat-mediated *FMR1* gene silencing seen in FXS and human cell-based/organoid models that can be used to verify the compounds and approaches that work in the animal models. In addition, the identification of molecular biomarkers, a focus area for FXS, will be useful not only to test the efficacy of treatment strategies based on restoring FMRP expression but also for those aimed at compensating for its loss.
